# Regulatory T Cells in γ Irradiation-Induced Immune Suppression

**DOI:** 10.1371/journal.pone.0039092

**Published:** 2012-06-19

**Authors:** Hugh I. McFarland, Montserrat Puig, Lucja T. Grajkowska, Kazuhide Tsuji, Jay P. Lee, Karen P. Mason, Daniela Verthelyi, Amy S. Rosenberg

**Affiliations:** Division of Therapeutic Proteins, Center for Drug Evaluation and Research, U.S. Food and Drug Administration, Bethesda, Maryland, United States of America; Tulane University, United States of America

## Abstract

Sublethal total body γ irradiation (TBI) of mammals causes generalized immunosuppression, in part by induction of lymphocyte apoptosis. Here, we provide evidence that a part of this immune suppression may be attributable to dysfunction of immune regulation. We investigated the effects of sublethal TBI on T cell memory responses to gain insight into the potential for loss of vaccine immunity following such exposure. We show that in mice primed to an MHC class I alloantigen, the accelerated graft rejection T memory response is specifically lost several weeks following TBI, whereas identically treated naïve mice at the same time point had completely recovered normal rejection kinetics. Depletion *in vivo* with anti-CD4 or anti-CD25 showed that the mechanism involved cells consistent with a regulatory T cell (T reg) phenotype. The loss of the T memory response following TBI was associated with a relative increase of CD4+CD25+ Foxp3+ expressing T regs, as compared to the CD8+ T effector cells requisite for skin graft rejection. The radiation-induced T memory suppression was shown to be antigen-specific in that a third party ipsilateral graft rejected with normal kinetics. Remarkably, following the eventual rejection of the first MHC class I disparate skin graft, the suppressive environment was maintained, with markedly prolonged survival of a second identical allograft. These findings have potential importance as regards the immunologic status of T memory responses in victims of ionizing radiation exposure and apoptosis-inducing therapies.

## Introduction

Ionizing radiation exposure results in a range of DNA damage including strand breaks, base damage, and crosslinking, which in turn induces apoptosis in radiation sensitive tissues including lymphocytes [1]. Immune suppression is a serious and immediate concern for victims of sublethal ionizing radiation exposure, such as became apparent following Hiroshima and Chernobyl, in that those exposed exhibited long term alterations in the composition of peripheral lymphoid populations and life-long impairment of immune responses [2], [3]. Atomic bomb survivors showed overall decreases in naïve T cell subsets, with normal CD4+, and increased CD8+ memory T cell populations [3]. In bulk culture, T cell responses to mitogens and alloantigens including IL-2 production and proliferation were reduced [1], [4], [5], attributable to the decreased proportion of CD4+ naïve T cells. Moreover, limiting dilution analysis revealed a decrease within the CD4+ T cell population of individual CD4+ T cells able to proliferate in response to mitogens and IL-2 or to produce IL-2 [6]. This may have resulted from direct radiation-induced genetic damage as well as to generation of an imbalance in T reg vs. T effector populations. Indeed, except for a single report which indicated an increase in putative T regs (CD4+CD25+) cells in individuals exposed to irradiation at Chernobyl [7], the function of regulatory T cells has not been examined in survivors of ionizing radiation. Preferential survival of Tregs relative to T effectors following sublethal γ irradiation could have profound effects on the composition and function of T cell populations for a prolonged time after exposure [8], [9], [10]. It is interesting to note that T regs also accumulate in aged humans [11] and mice [12] and that this has been associated with generalized impaired immune function [10]. Indeed, the alterations in T cell populations observed in victims of ionizing radiation exposure, including increased memory and T reg subsets, have been described as similar to the effects of aging [13], [1]. Thus, selective inhibition or depletion of adaptive T regs could potentially address the immunologic lesions observed both in aging and following γ radiation exposure.

In these studies, we examined the fate of the memory T cell response following sublethal γ irradiation, using an allograft rejection model in which CD8+ CTL are the effectors [14], [15]. Our study addressed induction of memory effector T cells and memory antigen specific T regs following priming, their fates following sublethal γ irradiation, and how our findings potentially pertain to the fate of vaccine immunity following sublethal γ irradiation.

## Results

### CD8+ T Cell-mediated Rejection of MHC Class I D^d^ Allografts by FVB Mice

To better understand the effects of ionizing radiation on memory T cell responses, we first identified the T cell populations required for rejection of D^d^ skin grafts in naïve and alloantigen primed FVB mice. As shown in Table 1, CD4+ T cell depletion had no effect on either primary or memory (accelerated) graft rejection responses, while CD8+ T cell depletion eliminated both primary and accelerated rejection responses. The results indicate that CD8+ T cells are both necessary and sufficient to reject MHC class I disparate D^d^ skin allografts in naïve mice and for accelerated rejection in antigen primed mice. In contrast, CD4+ T cells are neither necessary nor sufficient for allograft rejection across this antigenic disparity.

**Table 1:** Rejection of allogeneic D^d^ skin grafts by naïve and D^d^ primed FVB (H-2^q^) mice.

D^d^ Priming	Antibody Treatment	MST (d)
(−)	Control	14
(−)	anti-CD4	14
(−)	anti-CD8	>100*
(−)	anti-CD4+ anti-CD8	>100*
(+)	control	7
(+)	anti-CD4	7
(+)	anti-CD8	14*
(+)	anti-CD4+ anti-CD8	19*

In vivo depletion of CD4 and/or CD8 T cells. On days −6, −5, −1 and +5, GK1.5 (150 µg) mAb and the 53.6.7 (200 µg) mAb were injected IP into adult thymectomized FVB/N mice.

* Significant difference from control.

### Ionizing Radiation-induced T Memory Suppression

**Figure 1:**
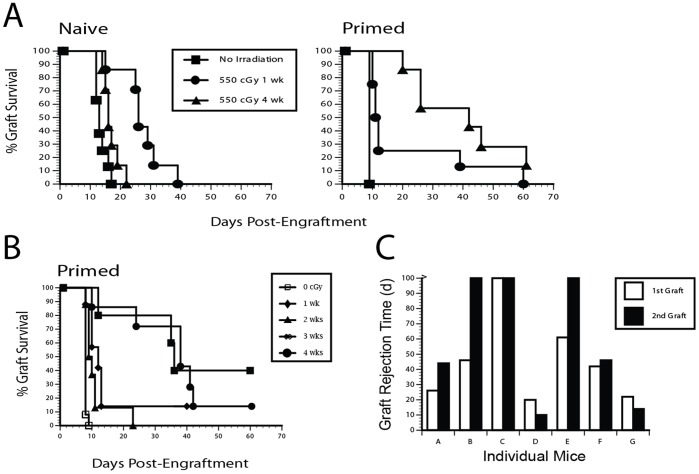
Allograft survival is prolonged in alloantigen primed, but not naïve mice, several weeks following γ-irradiation. A. Groups of 6 FVB mice were immunized IP with 2×10^7^ 3604 (D^d^) splenocytes, and 3 wks later given a 550 cGy dose of total body γ radiation (TBI). Mice were engrafted with 3604 tail skin grafts at either 1 or 4 wks following irradiation. B. Radiation induced T memory suppression (RITMS) develops between 2 and 3 weeks following γ-irradiation of immunized mice. Experiment designed as in A, except that separate groups of mice received allogeneic tail skin grafts at 1 or 2 or 3 or 4 weeks post-irradiation (1 wk vs. 3 wks: p<0.01, 2 wks vs. 3 wks. p<0.05, 1 or 2 wks vs. 4 wks, p<0.01, 1 vs. 2 wks, p<0.05). C. Suppression persists after eventual allograft rejection in RITMS mice. Mice from the experiment described in Fig. 1B were given a second identical ipsilateral allograft after the initial graft had rejected (77 d after the initial engraftment). Time to graft rejection is shown for individual mice. One mouse identified as “C” had 30% of the original allograft remaining. This original graft rejected after 149 days. The experiment was terminated after 100 d of observation of the second allograft.

To evaluate the effects of ionizing radiation on memory T cell mediated allograft rejection responses, we evaluated the time to rejection of an MHC class I disparate skin graft in antigen primed mice, as well as in control naïve mice, at time points following sublethal irradiation. Engraftment of naïve FVB mice with D^d^ skin grafts one week following sublethal γ irradiation (550 cGy) showed an expected prolongation of graft survival (MST 25 d vs. 12 d for control unirradiated mice, p<0.01), whereas engraftment of naive mice 4 weeks following irradiation revealed a near normalization of rejection times, indicating significant recovery of the cell populations required for allograft rejection (Figure 1A). Strikingly and reciprocally, engraftment of D^d^ primed FVB mice one week following irradiation resulted in an accelerated rejection response (7/9 mice)(Figure 1A), while engraftment of D^d^ primed mice 4 weeks after irradiation, resulted in markedly prolonged graft survival (MST 41 days), with several animals bearing intact grafts beyond the 100 day observation period. We termed this phenomenon “Radiation Induced T Memory Suppression” (RITMS). We then assessed the time course of development of RITMS. D^d^ primed mice allografted at 3 or 4 wks after irradiation showed significantly prolonged graft survival (MST 36, 37 d respectively) as compared to animals receiving allografts at 1 or 2 wks after irradiation (MST 10, 12 d respectively) (Figure 1B), indicating that development of RITMS required a minimum of 3 weeks following irradiation.

Although RITMS prevented acute graft rejection and robustly prolonged graft survival, most allografts eventually succumbed to chronic rejection. To determine whether RITMS persisted beyond the rejection of the first D^d^ skin graft, we regrafted RITMS mice from the experiment shown in Figure 1B with identical D^d^ disparate allografts 77 d after the initial skin graft, when 7/8 of the mice had fully rejected their allografts. As shown in Figure 1C, in mice demonstrating RITMS, immune suppression was retained even after rejection of the initial allograft and strikingly, in some mice, survival time for the second graft exceeded that of the first. In the two mice in which RITMS failed to develop initially (Figure 1C, mice D and G), accelerated rejection times, relative to the survival of the first graft were observed, indicating induction of a robust T effector memory response. Thus, in individual animals, whether the response to the second allograft was prolonged survival or rejection, the second response was of equal or greater magnitude than the first response, demonstrating an enduring suppressive or immune environment.

**Figure 2:**
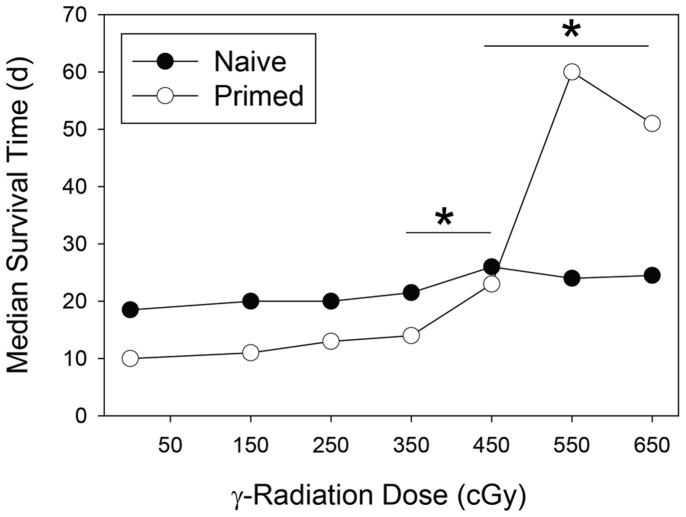
RITMS is generated within a discrete γ radiation dose range (450–650 cGy). Groups of 6 FVB mice were immunized with 2×10^7^ syngeneic (FVB) or allogeneic (3604) spleen cells IP. Three weeks later they received a single dose of γ radiation from 0 to 750 cGy (TBI), and were engrafted with 3604 allogeneic tail skin 4 weeks later.

We next evaluated the level of irradiation required for induction of RITMS. As shown in Figure 2, RITMS is induced at high, but sublethal doses of γ radiation, with the significant loss of accelerated rejection beginning at 450 cGy and peaking at 550–650 cGy (Figure 2). Thus, the RITMS effect on allograft rejection is optimal when mice are primed, receive a high, but sublethal dose of γ radiation followed by a several week time period prior to engraftment.

### Antigenic Specificity of RITMS

Because sublethal ionizing radiation is known to cause generalized immune suppression by apoptotic destruction of peripheral immune cells, we assessed whether the RITMS effect is antigenically specific. FVB mice were primed with H-2D^d^ disparate splenocytes (MHC-D^d^), control FVB, or third-party DBA/1 splenocytes which differ in multiple minor-H antigens from FVB, but bear the same MHC (H-2^q^). As shown in Table 2, prolonged survival of D^d^ disparate skin allografts on FVB mice following sublethal γ irradiation only occurred when the mice had been primed with D^d^ disparate spleen cells and not with syngeneic FVB or third party multiple minor mismatched DBA/1 spleen cells. The demonstration of enhanced survival of a D^d^ disparate skin graft but normal rejection of a DBA/1 skin graft on the same D^d^ primed irradiated mouse demonstrates that RITMS is antigen specific.

**Table 2:** Specificity of RITMS.

			Skin Graft MST (d)	
Recipient Strain	Priming Strain	Radiation Dose (cGy)	D^d^	DBA/1
FVB	D^d^	0	10	12
FVB	FVB	0	14	10
FVB	DBA/1	0	14	10
FVB	D^d^	450	33*	13
FVB	FVB	450	18	13
FVB	DBA/1	450	18	11

Groups of 10 mice were primed I.P. with 2×10^7^ syngeneic (FVB), multiple minor allogeneic (DBA/1), or MHC-D^d^ allogeneic (3604) spleen cells. γ radiation (550 cGy) was given 5 wks later, and mice were engrafted ipsilaterally with both DBA/1 and D^d^ disparate skin after an additional 6 wks.

### T Regs Proportionally Increase following Sublethal γ Irradiation

**Figure 3:**
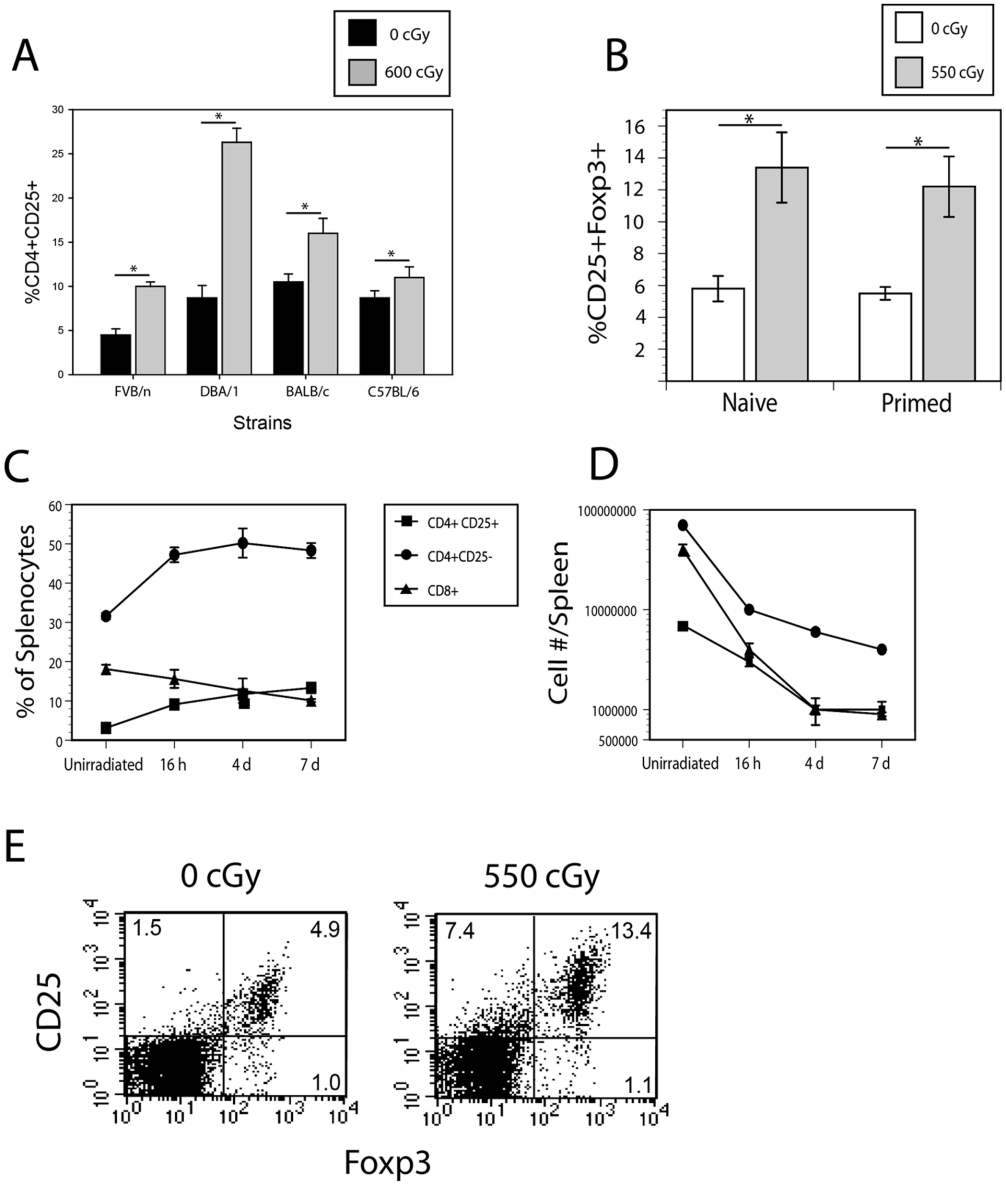
Tregs (CD4+CD25+Foxp3+) preferentially survive sublethal γ irradiation. (A) Spleen cells were obtained from groups of 3 FVB, DBA/1, BALB/c, and C57BL/6 mice. Samples were taken 7 d after 600 cGy TBI, and evaluated by flow cytometry for CD4 and CD25 expression. Data are presented as the percent of CD4+ cells expressing CD25. Error bars represent standard deviation of the mean. (B) LNC from groups of 6 FVB mice treated as in (A). Cells were stained for CD4, CD25, and Foxp3 and analyzed by flow cytometry. Data are presented as the percent of CD4+ cells expressing both CD25 and Foxp3. Error bars are S.D. and “*” represent statistical significance by t-test with p<0.01. (C) Relative increase in splenic Tregs and decrease in CD8+ T cells in the week following sublethal γ irradiation. FVB mice were given 550 cGy TBI and individual spleens were taken at the indicated intervals for flow cytometry analysis. Data are presented as the mean ± S.D. for groups of 3 mice. Data from this experiment are also presented in (D) as cell number per spleen. (E) Tregs present following sublethal γ-irradiation have increased expression of CD25 and Foxp3. Representative flow cytometry profiles of naïve (syngeneically primed) mice from (B). Numbers in each quadrant are the percentage of CD4+ cells expressing the indicated markers. Results were indistinguishable for allogeneically primed mice.

**Figure 4:**
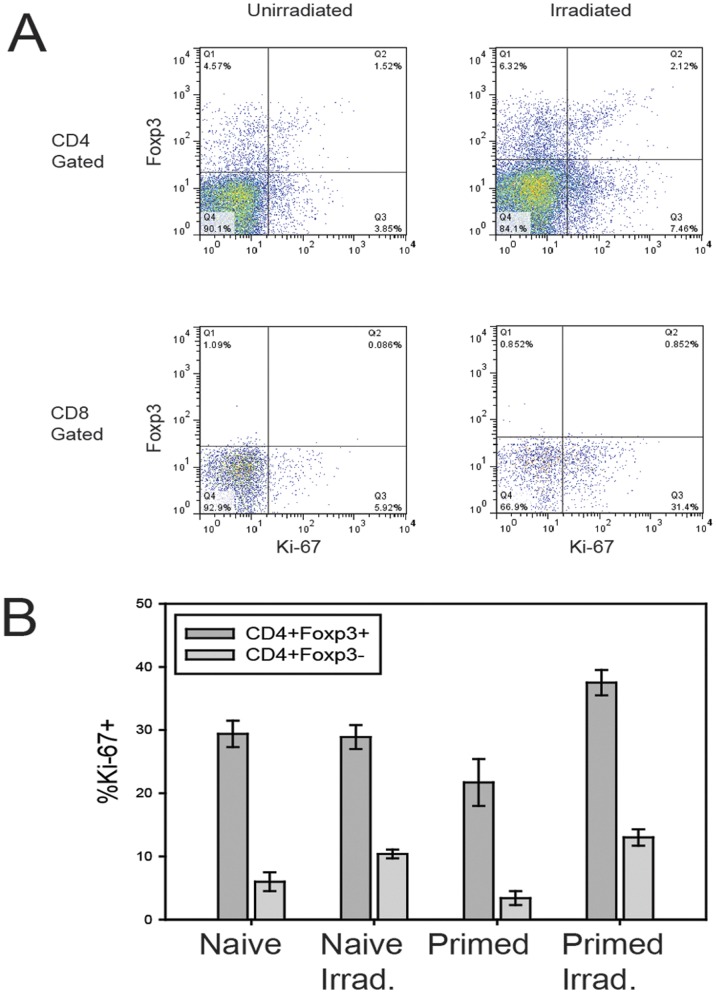
Increased proportion of T regs in cell cycle. Groups of 5 FVB/N mice were primed IP with 2×10^7^ 3604 or FVB/N splenocytes. Four weeks later the mice were exposed to 550 cGy TBI and 11 days later spleens were harvested. (A) Representative plots of splenocytes from unirradiated and irradiated mice stained for expression of CD4 or CD8 and Foxp3 and Ki-67. (B) The percentage of Ki-67+ cells among CD4+Foxp3+ and CD4+Foxp3**−** cell populations, showing naïve and D^d^-primed mice with and without irradiation. For each treatment group, a significantly greater proportion of the CD4+Foxp3+ cells express Ki-67 (p<0.001) than do the CD4+Foxp3**−** cell population.

The requirements for priming, a three week time interval following irradiation and the finding of antigen specificity suggested that an evolving cellular process was responsible for RITMS and led us to consider the possibility that this involved the development and activity of a suppressor or regulatory T cell population. Assessment of the T cell populations following sublethal γ radiation showed that all T cell populations were reduced in the 7 days following radiation. However, the reduction in CD4+ cells expressing CD25 and Foxp3, the prototypical surface phenotype of T regs, was less profound, resulting in a relative increase in this subpopulation with respect to other CD4+ and CD8+ T cell populations (Figure 3A–C). Prior immunization with alloantigen did not affect the magnitude of this increase (Figure 3B). The increase in the fraction of CD4+CD25+ splenocytes was evident as early as 16 hours following exposure to 550 cGy of γ irradiation, and continued to increase up to 4 days post-irradiation, at which point the ratio of CD8+ T cells to CD4+CD25+ T cells dropped from approximately 10∶1 to 1∶1 (Figure 3 C–D). In terms of absolute numbers, the loss of CD4+CD25− cells was approximately 3-fold greater than the loss of CD4+CD25+ cells (Figure 3D). The alteration in the ratio of effector CD8+ T cells to regulatory CD4+ T cells may be critical to the induction of RITMS, as CD8+ T cells are solely responsible for acute rejection of D^d^ skin grafts on FVB recipients (Table 1), and their reduction likely crucial to abrogation of acute graft rejection. The observed difference in the ratio of T regs to T effectors could potentially be due to increased resistance to apoptosis induced by *γ* irradiation and/or by enhanced proliferation. We did not find differences between CD4+Foxp3+ cells as compared to CD4+Foxp3− cells in anti-apoptotic Bcl-2 or Bcl-x_L_, or pro-apoptotic Bax molecules by flow cytometry (Figure S1). However, we did find a difference in proliferative capacity. As Ki-67 is strictly expressed during the cell cycle [16], we compared Ki-67 expression between CD4+Foxp3+ and CD4+Foxp3− cell populations and found a significantly higher proportion of CD4+Foxp3+ cells to have been in cell cycle in both unirradiated mice and in mice within 10 days of γ irradiation, as compared to CD4+Foxp3− cells (Figure 4). Of note, the relative increase in size of the T reg population was accompanied by phenotypic changes in this population only in the irradiated mice, namely increased fluorescence intensity in both CD25 and Foxp3 (Figure 3E) which correlates with enhanced function of such cells [17].

### *In vivo* Depletion with Anti-CD4 or Anti-CD25 Ablates RITMS

**Figure 5:**
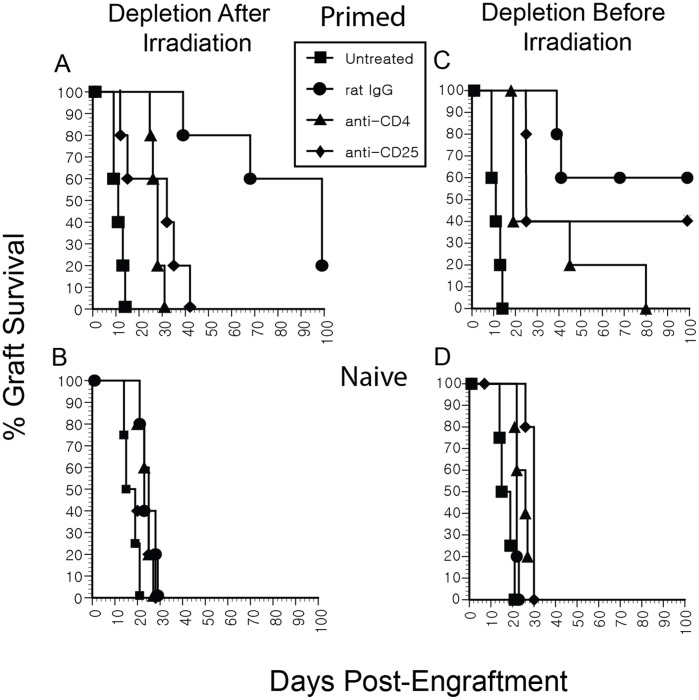
In vivo depletion with anti-CD4 or anti-CD25 ablates RITMS. One day after (A,B), or 2 wk before (C,D), 550 cGy TBI, groups of 6 D^d^ primed (2×10^7^ 3604 spleen cells IP 3 wks prior to TBI) or naïve (equal vol. PBS IP) FVB mice were given a single IP injection of 80 µg of anti-CD4 (GK1.5) or anti-CD25 (PC61) or control rat IgG. Mice in the “untreated” group received no irradiation or antibody treatment. All groups were engrafted with 3604 (D^d^) tail skin 4 weeks after irradiation. At 10 d after mAb treatment CD25 and CD4 depletion were >90%. Similar results were obtained in two analogous depletion experiments, one of which used the rat IgG2a GL113 as an isotype control for PC61.

To investigate the mechanism of RITMS, T regs were depleted *in vivo* with anti-CD25 mAb (PC61), or with anti-CD4 mAb (GK1.5) either 2 weeks prior to or 1 d following TBI. As shown in Figure 5A and C, allograft survival times in RITMS mice were significantly restored by eliminating T regs (p<0.05 for administration of depleting mAbs before or after irradiation, and for both anti-CD4 and anti-CD25 mAbs compared to rat IgG), supporting the hypothesis that these cells play a key role in the induction of RITMS. No effects of depletion were observed in irradiated naive mice (Figure 5 B and D), again emphasizing the requirement for prior antigen exposure to generate T regs capable of mediating RITMS.

### Allograft Survival is Associated with a Shift in the Levels of Foxp3:CD8 mRNA in Skin Grafts

**Figure 6:**
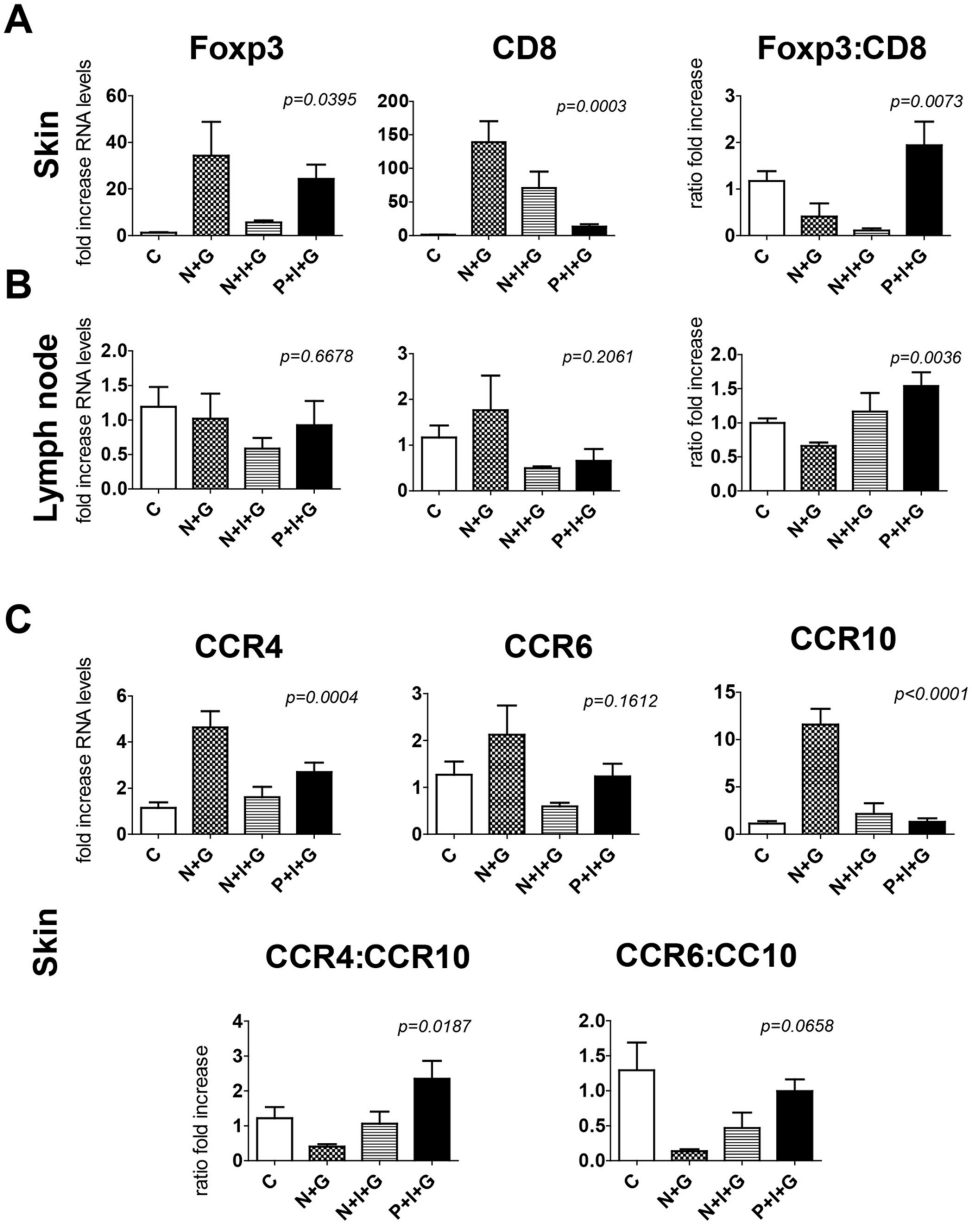
Expression of Foxp3, CD8 and skin-homing chemokine receptor genes in allogeneic skin grafts and lymph nodes. D^d^ primed (**P**) or unprimed (naïve, **N**) mice were treated with 550 cGy of γ irradiation (**I**) and subsequently received 3604 tail skin grafts (**G**) 21 days after irradiation. Allografts and LN were harvested at 16 days post-engraftment, when (data not shown) primed and unirradiated primed mice had rejected their allografts. Fold increase and ratio of fold increase were compared for Foxp3 and CD8 mRNA for skin (Figure 6A), and lymph nodes (Figure 6B), and for chemokine receptor expression in allografted skin (Figure 6C) by real-time PCR (n = 4−6/group) relative to control untouched skin (**C**). Statistical significance between groups is indicated in each graph and as assessed by One Way ANOVA and Tukey’s multiple comparison test.

The finding that T regs were critical to RITMS prompted us to evaluate the T reg content of the allografts themselves following irradiation. Thus, allografts were harvested from RITMS mice and controls at day 16 post engraftment for assessment of Foxp3 and CD8 (cell subset markers) as well as skin-homing markers (CCR4, CCR6 and CCR10) by real-time PCR analysis of total mRNA The expression of these markers in allografts was compared to that in the draining lymph nodes (LN) of the same animals. In the skin allografts of naïve unirradiated animals, we observed a significant increase in the levels of transcripts of all the markers tested, in particular in CD8 (Figure 6A) and CCR10 (Figure 6C) expression. As expected, the expression of these genes was reduced in the two experimental groups of mice that were irradiated. However, while the reduction affected CD8 and Foxp3 expression in the naïve-irradiated group, proportionately to that in unirradiated allografts, allografts from D^d^ primed mice showed a very low level of CD8 RNA, but sustained Foxp3 RNA levels. This resulted in a dramatic shift in the ratio of Foxp3 to CD8 RNA (Figure 6A) that favors T regs. Unlike skin, LN did not show any significant changes in the levels of RNA of these markers (Figure 6B). Cell infiltration was assessed by identifying changes in expression of chemokine receptor genes associated with cells that are trafficking to the skin, such as CCR10 (in CD8+ effector T cell subsets [18]) and CCR4 and CCR6 (in lymphocytes including T regs [19], [20]) (Figure 6C). The ratio of fold increase in CCR4:CCR10 and CCR6:CCR10 was found to be inverted in the primed/irradiated mice compared to the other two groups (Figure 6C), strongly correlating with the increased ratio of Foxp3:CD8 RNA. These data associate allograft survival with a marked decrease in infiltration of CD8+ cells into the allograft relative to Foxp3 expressing cells.

## Discussion

### Ionizing Radiation Induced Immune Suppression

Our studies demonstrate that following sublethal irradiation of antigen-primed mice, antigen-specific immune suppression develops over a several week time period and is mediated primarily by CD4+CD25+ T regs. This antigen-specific suppression requires priming, a radiation dose of 550–650 cGy, and a minimum time period of three weeks following irradiation prior to challenge (Figure 1). The reason for this several week delay is not clear but may include the requirement for relative expansion of the T reg population during radiation-induced lymphopenia, and any effects of lymphopenia driven proliferation (LDP) on expression of suppressor function. Alternatively, it may reflect the time required for the generation of T regs from precursor populations.

Suppression of acute graft rejection resulted in development of a chronic rejection response and the loss of the graft over a prolonged time period. However, despite eventual loss of most first grafts due to chronic rejection, subsequent skin grafts on the same mice enjoyed survival times at least as long as those of the first grafts, with some appearing to be fully accepted, thus indicating a sustained suppressive environment (Figure 1C). The cellular basis for this suppression appears to be enhanced survival of adaptive memory-like T regulatory cells, and their dominance over CD8 effector cells following sublethal irradiation and LDP. Our studies of the RITMS model suggest that MHC class I D^d^ specific CD4+CD25+ T regs require antigenic priming, that they survive irradiation, that they migrate into the allograft, and that they maintain a long term “suppressive” environment with respect to the specific antigen.

### The Importance of Priming in RITMS

Several observations support the critical role of priming in the generation of CD4+CD25+ T regs capable of suppressing acute rejection responses. First, although no differences were observed in the number of T regs between naïve and alloantigen-primed mice in the RITMS model, prolonged allograft survival was only evident in antigen primed mice. Second, depletion of CD25+ T cells following priming, but before irradiation and challenge substantially abrogated the RITMS effect of prolonging graft survival (Figure 5). These results concur with those of Moxham et al. [14] which showed that although Foxp3+CD4+ T cells were present at high levels in kidney allografts of RAG mice that received naïve T cells, such allografts received no apparent protection from acute rejection. Thus, unlike the RITMS phenomenon, in which antigen primed T regs ably suppressed rejection of allografts, not only for one allograft in the short term, but over successive grafts over a longer time frame, the naive T regs in both the Moxham work, as well as in our own, failed to suppress graft rejection despite their increased presence in the allograft.

Having demonstrated the role of T regs in RITMS by *in vivo* depletion, we chose to examine the levels of CD8 and Foxp3 transcripts in the allografts by real-time PCR. As this method cannot rule out whether the increase in RNA in the tissue is due to cell proliferation or infiltration, we also evaluated the expression of genes of chemokine receptors present in cells that are homing to the skin, such as CCR4, CCR6 and CCR10.

In naive unirradiated mice, real-time PCR data suggested an influx of CD8+ T cells and T reg cells into allografts (Figure 6 A–C), characterized by a shift in the T cell subset markers in concordance with associated chemokine receptor markers: CCR10 for CD8+ T cells (18) and CCR4 and CCR6 for T regs (19,20). Gamma-radiation exposure decreased the influx of both CD8+ T cells and T regs into the allografts, although more markedly for the CD8+ T cells of primed/allografted mice. Foxp3 RNA levels were maintained when the animals were primed prior to irradiation, almost at the levels observed in naïve unirradiated mice. This finding parallels the profile in blood and secondary lymphoid organs showing an alteration in the CD8+**:**CD4+CD25+ ratio (Figure 3C) in irradiated vs. unirradiated animals. However, we have demonstrated that only in alloantigen-primed mice do we see prolongation of allograft survival (Figure 1, 2). The likeliest explanation for this, in agreement with the work of Dai et al. [15], is that even though T regs from naïve mice migrate selectively into allografts, only memory-like alloantigen-experienced T regs inhibit the activity of memory alloantigen-specific CTL. Memory-like T regs may be effective where naïve T regs fail because, as with other memory cellular responses, the responses of memory-like T regs may be accelerated and the frequency of antigen specific T regs in the total T reg population is likely increased relative to the frequency found in naïve T cell populations.

While D^d^ primed T regs potently suppressed D^d^ skin graft rejection, they had no effect on the rejection kinetics of a third-party allograft, demonstrating the antigenic specificity of the suppression: DBA/1 skin graft survival following sublethal γ irradiation was not prolonged either by DBA/1 or D^d^ priming (Table 2). The lack of prolonged survival of the DBA/1 skin graft on the DBA/1 primed mice may be secondary to the lower dose of γ irradiation used (450 cGy), to different kinetics of the memory response to multiple minor antigens vs. a single MHC class I alloantigen or to a more robust overall immune response involving different T cell helper and effector cells [21]. Indeed, the rejection response of FVB mice primed to DBA/1 was only one day faster than that of naïve mice. This is currently under investigation.

### Skewing of T Reg to T Effector Ratio

For both irradiation-exposed primed and naive mice, the proportion of T regs to T effector populations is skewed to relatively higher numbers of T regs (Figure 3). Similar alterations in T reg populations have been noted following ionizing radiation exposure in other animal models, [22], [23], [24], and the expanded T regs have been found to suppress normally *in vitro* [25]. This alteration in the size ratio of T regs and other T cell populations may be due to the differential susceptibility to γ radiation-induced apoptosis or to differential rates of proliferation. Increased Bcl-2 expression has been reported in CD4+CD25^high^ cells as compared to CD4+CD25− cells in mice [24], [26] suggesting enhanced resistance to γ radiation-induced apoptosis. We did not observe a difference in Bcl-2 levels in these populations (Figure S1) possibly due to strain or substrain differences in expression. No difference in Bcl-2 has been noted between CD4+CD25+ cells and CD4+25− cells in irradiated human PBL [27], [28]. However, humans who have been exposed to sublethal ionizing radiation show similar changes in the ratio of T regs to CD4+ T effectors [7], [29], suggesting that radioresistance mediated by Bcl-2 may not be the mechanism of the altered ratio in human populations. Here we provide evidence for increased proliferation of CD4+Foxp3+ cells over CD4+Foxp3− cells, in both non-irradiated, as well as irradiated animals by flow cytometric measurement of Ki-67 (Figure 4). Surprisingly, radiation did not enhance Ki-67 expression as would be expected with lymphopenia-driven proliferation (LDP), suggesting that these cells are maximally proliferating even in the lymphocyte replete host. An increased turnover of T reg phenotype cells as compared to CD4+Foxp3− cells as measured by Ki-67 staining has been reported as well in human PBL [30]. Supporting a role for enhanced proliferation in RITMS, human T regs have been shown to have a competitive advantage in LDP as compared to effector T cells [31], [32]. Thus, it is possible that more robust proliferation of CD4+Foxp3+ Tregs over their CD4+Foxp3+ counterparts (as may occur because of enhanced uptake of IL-2, consistent with their higher expression of CD25), as well as a significant decrease in CD8 effector cell numbers, averted acute rejection responses and engendered a sustained suppressive environment [25]. Aside from increased proliferation of CD4+CD25+ T regs, it is also possible that “quiescent” Tregs, which express Foxp3 but not CD25, upregulate CD25 following irradiation, thereby improving their survival and contributing to the CD4+CD25+Foxp3+ Treg pool [33]. An additional possibility is that allospecific memory CD4+ T helper cells are converted to allospecific T regs by γ irradiation and the resultant LDP and alterations in the cytokine milieu [34]. This possibility is under investigation.

In a number of studies we attempted to examine the function of the memory-like T regs that mediate RITMS, but were unable to show prolongation of allograft survival following *in vivo* administration of T regs from RITMS mice. Qu et al. (25) demonstrated that T regs enriched after irradiation had suppressive function in vitro. Our chief impediment may be the rarity of memory-like T regs in the much larger pool of T regs of other specificities.

### Ionizing Radiation Exposure and Vaccine Immunity

These studies showed that sublethal ionizing radiation exposure results in suppression of memory responses both by apoptotic destruction of memory T effector cells, but also by the enhanced survival, proliferation and activity of memory-like T regs. We hypothesize that the phenotypic hallmarks of memory T regs are enhanced expression of Foxp3 as well as CD25, relative to naïve T regs. While this process may be important to reduce damage to the immune system immediately following irradiation, it may impair responses to infectious agents by suppressing the activation of memory responses accrued through years of exposure and vaccinations. Improved understanding of the effects of sublethal radiation on T cell homeostasis may allow the necessary manipulations to prevent suppression of vaccine immunity following exposure to sublethal γ irradiation. Such studies are underway.

## Materials and Methods

### Mice and Priming

FVB mice (H-2^q^) were purchased from Taconic Farms (Germantown, NY) and DBA/1 (H-2^q^) mice were obtained from The Jackson Laboratory (Bar Harbor, ME). Strain 3604 (MHC-D^d^) transgenic mice were generated as described [35], expressing H-2D^d^ using the endogenous MHC promoter resulting in D^d^ expression by all lymphocytes and on skin. Priming (immunization) was achieved with a single IP dose of 2×10^7^ MHC-D^d^ splenocytes. Mice designated as “unprimed” received a single IP dose of 2×10^7^ syngeneic FVB splenocytes. “Naïve” control mice were given an equal volume (200 µL) of PBS IP. Mice were housed in a specific pathogen-free animal facility, and all animal experiments were approved by the Center for Biologics Evaluation and Research (CBER) or White Oak Institutional Animal Care and Use Committees in strict accordance with the recommendations in the Guide for the Care and Use of Laboratory Animals of the National institutes of Health. OLAW Assurance #A4295-01.

### Irradiation

Total body γ irradiation was performed using a Gammacell 40 Cs-137 irradiator (MDS Nordion, Mississauga, ON) with a dose rate of 65 cGy/min.

### Skin Grafting

Mouse tail skin was engrafted on the flank of recipient mice as described [36]. Grafts were scored daily and were considered rejected when 20% or less of engrafted tissue remained.

### Flow Cytometry

Flow cytometric analysis was performed on a FacsCalibur (Becton Dickinson [BD]) and analyzed using Cell Quest (BD) or Flowjo (Tree Star, Inc). The following antibodies were obtained from BD: CD8 (53-6.7), CD4 (GK1.5), CD25 (PC61), CD16/CD32 (2.4G2). Bcl-2 (10C4) and Ki-67 (SolA15) were obtained from eBioscience. Bcl-x_L_ (H-5) and and Bax (B-9) were purchased from Santa Cruz Biotechnology. Intracellular stainings were performed using Cytofix/Cytoperm (BD). Appropriate isotype controls were matched with each antibody.

### *In vivo* Depletion

The following mAbs were used for in vivo depletion studies: Rat IgG (SIGMA I4131), anti-CD4 (GK1.5, rat IgB2b, ATCC), anti-CD25 (PC61, rat IgG2a), anti–*Escherichia coli* ß-galactosidase (GL113, rat IgG2a a gift from Fred Finkelman, University of Cincinnati), anti-CD8 (53.6.7 ATCC).

### RNA Extraction and Gene Expression Analysis

Untouched skin or skin from grafts were removed from the mouse and immediately placed in 1 ml of TRIzol Reagent (Invitrogen Life Technologies) and kept at −80°C. One mm diameter Zirconia beads (Biospec Products, Bartlesville, OK) were added to the samples prior to homogenization in the MiniBeadBeater-8 (Biospec Products, Bartlesville, OK). Each sample was homogenized for 30 seconds, and then rested on ice for 90 seconds, for five consecutive times. Subsequently the samples were centrifuged for 15 minutes at 13,000 rpm. The TRIzol reagent was transferred to a new tube, and the RNA was extracted following manufacturer’s recommendations. Reverse Transcription of the RNA was done using High Capacity cDNA Reverse Transcription Kit (Applied Biosystems, Foster City CA) as per manufacturers’ instructions. Relative mRNA levels for Foxp3 and CD8 genes were analyzed using the corresponding TaqMan Gene Expression assay by real-time PCR (Applied Biosystems, Foster City CA). Values for the target gene were normalized using 18S RNA. Expression values were calculated using the 2^−ΔΔ*Ct*^ method [37] and expressed relative to the untouched skin control.

### Statistics

The Wilcoxon Signed-Rank test was used to compare skin graft survival data. Real time PCR data were analyzed using ANOVA and Tukey’s multiple comparison test. Student’s t-test was used to evaluate the significance of all other data.

## Supporting Information

Figure S1 **Expression of pro- and anti-apoptotic molecules by CD4+Foxp3+ and CD4+Foxp3− cells.** Spleens were harvested from 5 individual unmanipulated female FVB/N mice. Cells were stained for CD4, Foxp3, and either Bcl-2, Bcl-x_L_, or Bax, for flow cytometric analysis. Histograms show isotype controls (red), CD4+Foxp3− (yellow), and CD4+Foxp3+ (blue), stained with apoptosis markers as indicated.
(TIF)
