# Supplementary figures and images for: Regulatory T Cells in γ Irradiation-Induced Immune Suppression
Source: PLoS One. 2012 Jun 19;7(6):e39092. doi: 10.1371/journal.pone.0039092 (PMC3378522; doi:10.1371/journal.pone.0039092)

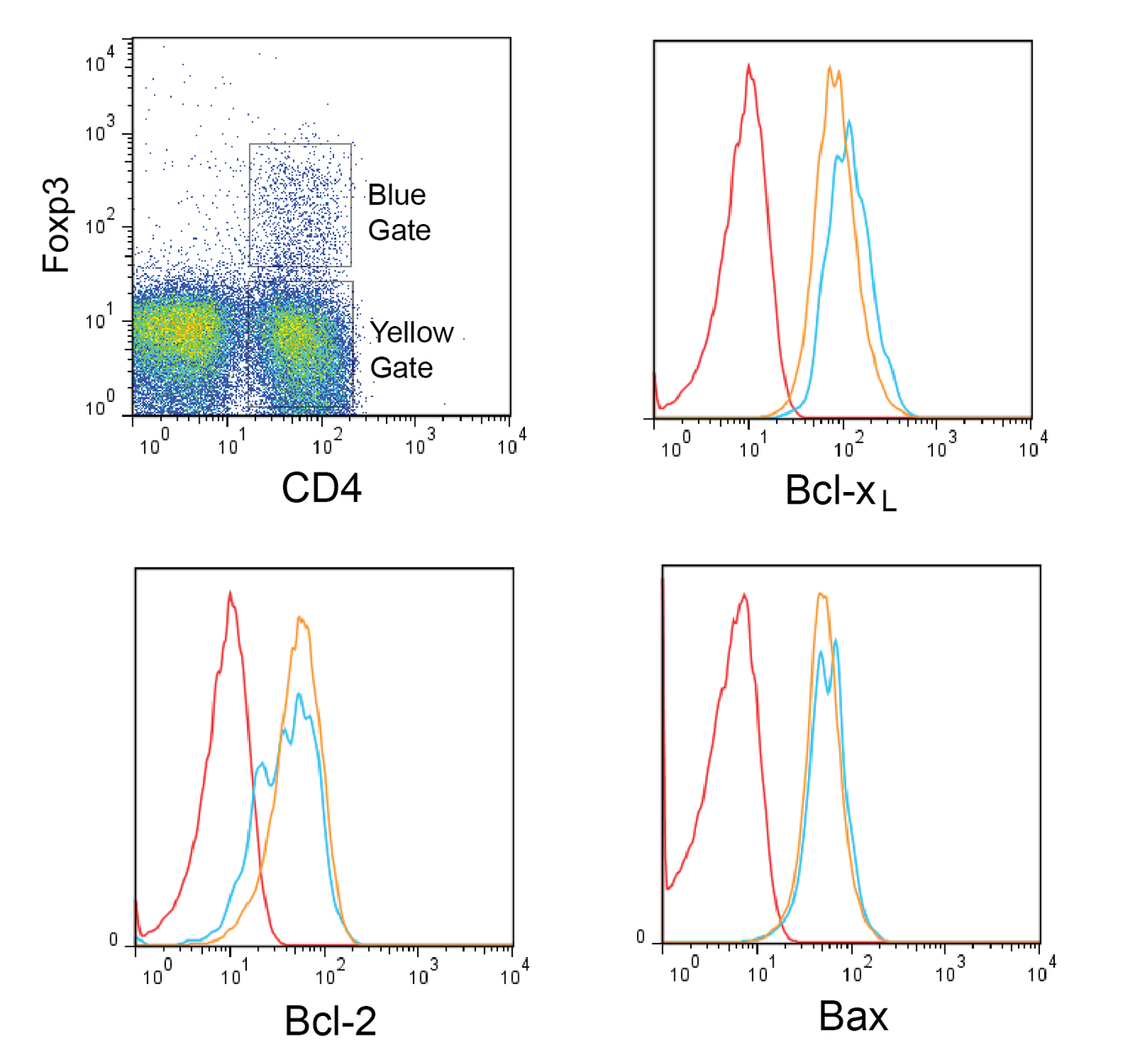

Supplement: Figure S1 — Expression of pro- and anti-apoptotic molecules by CD4+Foxp3+ and CD4+Foxp3− cells. Spleens were harvested from 5 individual unmanipulated female FVB/N mice. Cells were stained for CD4, Foxp3, and either Bcl-2, Bcl-xL, or Bax, for flow cytometric analysis. Histograms show isotype controls (red), CD4+Foxp3− (yellow), and CD4+Foxp3+ (blue), stained with apoptosis markers as indicated. (TIF) [file pone.0039092.s001.tif]
